# Development of risk-score model in patients with negative surgical margin after robot-assisted radical prostatectomy

**DOI:** 10.1038/s41598-024-58279-1

**Published:** 2024-03-31

**Authors:** Yuta Yamada, Yoichi Fujii, Shigenori Kakutani, Naoki Kimura, Kazuma Sugimoto, Yuji Hakozaki, Toru Sugihara, Yuta Takeshima, Taketo Kawai, Masaki Nakamura, Jun Kamei, Satoru Taguchi, Yoshiyuki Akiyama, Yusuke Sato, Daisuke Yamada, Fumihiko Urabe, Hideyo Miyazaki, Yutaka Enomoto, Hiroshi Fukuhara, Tohru Nakagawa, Tetsuya Fujimura, Haruki Kume

**Affiliations:** 1 Department of Urology, Graduate School of Medicine, The University of Tokyo, 7-3-1, Hongo, Bunkyo-Ku, Tokyo 113-8655 Japan; 2 Department of Urology, Chiba Tokushukai Hospital, Funabashi-Shi, Chiba Japan; 3 Department of Urology, National Center for Global Health and Medicine, Shinjuku-Ku, Tokyo Japan; 4 Department of Urology, Jichi Medical University, Shimotsuke-Shi, Tochigi-Ken Japan; 5 Division of Innovative Cancer Therapy, Advanced Research Center, The Institute of Medical Science, The University of Tokyo, Minato-Ku, Tokyo Japan; 6 Department of Urology, Teikyo University School of Medicine, Itabashi-Ku, Tokyo Japan; 7 Department of Urology, NTT Medical Center Tokyo, Shinagawa-Ku, Tokyo Japan; 8 Department of Urology, The Jikei University School of Medicine, Tokyo, Japan; 9 Department of Urology, Mitsui Memorial Hospital, Chiyoda-Ku, Tokyo Japan; 10 Department of Urology, Kyorin University School of Medicine, Mitaka, Tokyo Japan

**Keywords:** Robot-assisted radical prostatectomy, RARP, Negative surgical margin, Prostate cancer, Non-metastatic, Oncology, Urology

## Abstract

A total of 739 patients underwent RARP as initial treatment for PCa from November 2011 to October 2018. Data on BCR status, clinical and pathological parameters were collected from the clinical records. After excluding cases with neoadjuvant and/or adjuvant therapies, presence of lymph node or distant metastasis, and positive SM, a total of 537 cases were eligible for the final analysis. The median follow-up of experimental cohort was 28.0 (interquartile: 18.0–43.0) months. We identified the presence of International Society of Urological Pathology grade group (ISUP-GG) ≥ 4 (Hazard ratio (HR) 3.20, 95% Confidence Interval (95% CI) 1.70–6.03, P < 0.001), lymphovascular invasion (HR 2.03, 95% CI 1.00–4.12, P = 0.049), perineural invasion (HR 10.7, 95% CI 1.45–79.9, P = 0.020), and maximum tumor diameter (MTD) > 20 mm (HR 1.9, 95% CI 1.01–3.70, P = 0.047) as significant factors of BCR in the multivariate analysis. We further developed a risk model according to these factors. Based on this model, 1-year, 3-year, and 5-year BCR-free survival were 100%, 98.9%, 98.9% in the low-risk group; 99.1%, 94.1%, 86.5% in the intermediate-risk group; 93.9%, 84.6%, 58.1% in the high-risk group. Internal validation using the bootstrap method showed a c-index of 0.742 and an optimism-corrected c-index level of 0.731. External validation was also carried out using an integrated database derived from 3 other independent institutions including a total of 387 patients for the final analysis. External validation showed a c-index of 0.655. In conclusion, we identified risk factors of biochemical failure in patients showing negative surgical margin after RARP and further developed a risk model using these risk factors.

## Introduction

Prostate cancer (PCa) is the second most frequently diagnosed cancer and the fifth leading cause of cancer-related death among men worldwide^1^. Robot-assisted radical prostatectomy (RARP) has gained popularity in treating clinically localized PCa due to its excellent dexterity under the magnified view. Although RARP is an effective treatment in such patients, up to 13–19% of the patients will eventually experience biochemical recurrence (BCR) within 5–7 years after surgery^2^. Determination of factors associated with BCR is crucial for clinical practice such as patient follow-up and determination of adjuvant or salvage treatment.

However, identifying factors of BCR in patients undergoing RARP is relatively difficult since there are various relevant factors. Such factors include prostate-specific antigen (PSA) density, pathological Gleason score (GS), pathological T stage, positive surgical margin (SM), and others^2–4^. Among these factors, positive SM is one of the strongest factors of BCR^5^. In patients with positive SM, the oncologic status of the tumor at the site of positive SM may affect BCR. However, in patients with negative SM, tumors are completely resected en bloc with the prostate. Therefore, there may be different grounds for factors associated with BCR in patients between negative and positive SM. From this perspective, it is critical to mitigate the possible concerns influenced by this key factor. To resolve this concern, we considered that investigation of the factors of BCR should be conducted separately in patients with negative and positive SM. In this study, we investigated the predicting factors of BCR in patients with negative SM after RARP for non-metastatic PCa. We further established a novel risk model for predicting BCR, and performed internal and external validation of the model.

## Methods

### Patients and study design

**Figure 1:**
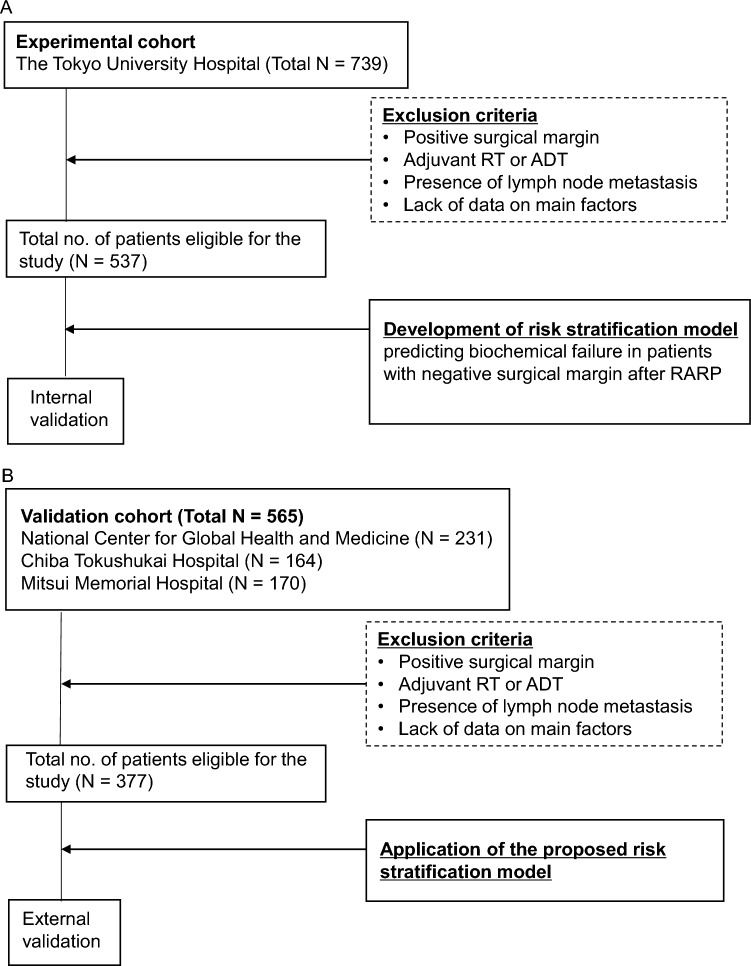
Flowcharts including the description of participants/collaborating institutions and methods of the study. A total of 537 and 377 patients were eligible for the final analysis in the experimental and validation cohorts, respectively. *RT* radiation therapy, *ADT* androgen deprivation therapy, *RARP* robot-assisted radical prostatectomy.

A total of 739 patients underwent RARP for prostate cancer (PCa) at Tokyo University Hospital from November 2011 to October 2018. Data on clinical and pathological parameters including BCR status, PSA, and adverse pathology (International Society of Urological Pathology grade group (ISUP-GG), extraprostatic extension, seminal vesicle invasion, lymphovascular invasion, perineural invasion, lymph node metastasis) were collected from the medical records. To analyze patients with negative SM in non-metastatic PCa, we excluded cases as follows. (1) Cases with positive SM and/or clinical and pathological lymph node metastasis, (2) patients who were treated with neoadjuvant and/or adjuvant therapy, (3) cases associated with lack of data on major factors such as BCR status of pathological parameters (Fig. 1A). After excluding cases based on these criteria, a total of 537 patients were eligible and used as an experimental cohort (Fig. 1A).

For external validation, a database was collected from 3 independent institutions and was integrated into one external cohort. Database of each institution was available for use from July 2016 to July 2021 at National Center for Global Health and Medicine, May 2017 to May 2019 in Chiba Tokushu-kai Hospital and Mitsui Memorial Hospital (Fig. 1B).

BCR was defined as 2 consecutive rises in PSA values above 0.2 ng/mL or the introduction of salvage treatment. Staging of PCa was performed using the TNM staging system proposed by the European Association of Urology (EAU)^6^. Standard follow-ups after RARP were carried out at our outpatient department after discharge at 2 weeks, 1, 3, 6, 12 months, and a 6–12 months cycle thereafter.

Written informed consent was not obtained from all study participants since ethical approval by the ethics committees “Research Ethics Committee of the Faculty of Medicine of the University of Tokyo” (ID: 2020039NI) of the University of Tokyo, “The ethical committee of Mitsui Memorial Hospital” (ID: 2020C30) of Mitsui Memorial Hospital, “The ethical committee of Chiba Tokushu-kai Hospital” (ID: 184) of Chiba Tokushu-kai Hospital, and “The ethical committee of National Center for Global Health and Medicine” (ID: 143) of National Center for Global Health and Medicine was granted for ‘opt-out’ consent to apply to the present cohort. This study was conducted under the Helsinki Declaration.

### Surgical method

RARP was performed by using the da Vinci surgical robot system (da Vinci-S, Si, or Xi ®: Intuitive Surgical Incorporation, Sunnyvale, CA) as described in our previous studies^7^. Briefly, the RARP procedure was carried out by a transperitoneal approach using 6 ports, 4 of which were for robotic arms. The nerve-sparing procedure was carried out in indicated cases based on the choice of the patient and the surgeon. The urethra was cut adjacent to the distal edge of the prostatic apex. The pelvic floor was repaired using Rocco’s stitch technique following the resection of the prostate^8^. Anastomosis of the urethra and bladder was carried out by a single-knot running suture of 3-0 absorbable monofilament^9^. Pelvic lymph node dissection included removal of the obturator, external, and internal iliac lymph nodes.

### Statistical analyses

Statistical analysis was carried out using the statistical software R version 4.1.0. Packages “survival”, “survminer”, and “rms” were used in this study. Kaplan–Meier curves were drawn to compare the time to PSA failure in the chosen groups and were statistically analyzed by log-rank test. Univariate and multivariate analyses using the Cox regression hazard model were performed to identify predicting factors of BCF in the experimental cohort. Internal validation was carried out using the bootstrap method. A P-value of < 0.05 was considered statistically significant.

### Model development and validation

We developed a novel risk stratification model to predict BCF in patients with negative surgical margins after RARP in non-metastatic PCa patients. This model was developed using the 4 robust risk factors associated with BCR according to the multivariate analysis (‘ISUP-GG ≥ 4’, ‘presence of lymphovascular invasion’, ‘presence of perineural invasion’, ‘MTD > 20 mm’). Weighted scores were given according to the coefficient value in the multivariate analysis and the total scores were calculated according to the rules as follows: 1 point was given if the value of the coefficient was 0–1, 2 points if the coefficient value was 1–2, and 3 points when it was over 2 (Fig. 3A). Three distinctive risk groups were observed according to the total scores. Low, intermediate, and high-risk groups were defined as cases with 0–2, 3–4, and 5–7 points, respectively (Fig. 3A). Internal validation was performed by evaluating the discrimination of the model by using the concordance index (c-index). The model was internally validated using a bootstrap method with 3000 bootstrap resamples and 1000 repetitions of calculating the c-index of the risk model in each bootstrap-resample set to assess the optimism necessary for calculating the optimism-corrected c-index (i.e. apparent c-index minus optimism).

The risk model was also externally validated using a validation cohort based on an integrated database of 3 independent institutions (Mitsui Memorial Hospital, Chiba Tokushu-kai Hospital, and National Center for Global Health and Medicine).

### Model communication

We also developed a 1, 3, and 5-year risk assessment nomogram for clinical use and to support the translation of the generated risk model (Supplementary Fig. 1A). Note that the nomogram was generated by the formula of the Cox hazard model based on the experimental cohort. The calibration plot was also performed for 1, 3, and 5-year risks (Supplementary Fig. 1B–D).

## Results

### Demographics of patients undergoing RARP for clinically non-metastatic prostate cancer

Demographics of the experimental and validation cohorts are shown in Table 1. There were 537 and 377 cases for the experimental and validation cohorts, respectively. The cohorts showed statistically different signatures in terms of age, distribution of biopsy Gleason score, clinical T (cT) stage, and prostate weight (Table 1). Specifically, the validation cohort included more cases with ≥ cT2 and also a higher biopsy Gleason score. The median follow-up of the experimental cohort was 28.0 (interquartile (IQR):18.0–43.0) months.

**Table 1:** Baseline characteristics of experimental and validation cohorts.

		Experimental cohort (internal cohort) (N = 537)	Validation cohort (external cohort) (N = 377)	P value
Median age, (IQR)		68 (63–71)	71 (67–76)	< 0.001
PSA, (IQR)		7.0 (5.0–10.0)	7.0 (5.0–10.0)	0.105
Biopsy ISUP-GG	1	107	57	< 0.001
	2 and 3	313	232	
	4 and 5	117	182	
	Unknown	0	1	
cT stage	cT1c	441	145	< 0.001
	≥ cT2	96	231	
	Unknown	0	1	
Prostate weight		40 (32–51)	48 (37–58)	< 0.001
Perineural invasion	Neg.	151	138	0.008
	Pos.	386	239	
Extraprostatic extension	Neg.	423	304	0.545
	Pos.	114	73	
Lymphovascular invasion	Neg.	320	288	< 0.001
	Pos.	217	89	
Seminal vesicle invasion	Neg.	517	355	0.180
	Pos.	20	22	
Max. diameter of tumor with any ISUP-GG (MTD)		17 (13–23)	16 (11–22)	0.065
Max. diameter of tumor with max. ISUP-GG (MTD-mGG)		16 (12–23)	15 (10–21)	0.005
ISUP-GG	1	28	27	0.101
	2 and 3	388	248	
	≥ 4	121	102	

cT stage was determined according to the EAU-EANM-ESTRO-ESUR-ISUP-SIOG guideline on prostate cancer [https://d56bochluxqnz.cloudfront.net/documents/pocket-guidelines/EAU-EANM-ESTRO-ESUR-ISUP-SIOG-Pocket-on-Prostate-Cancer-2022.pdf].

*SM* surgical margin, *IQR* interquartile range, *BMI* body mass index, *PSA* prostate specific antigen, *ISUP-GG* International Society of Urological Pathology grade group.

### Factors of biochemical recurrence based on univariate and multivariate analyses in the experimental cohort

**Figure 2:**
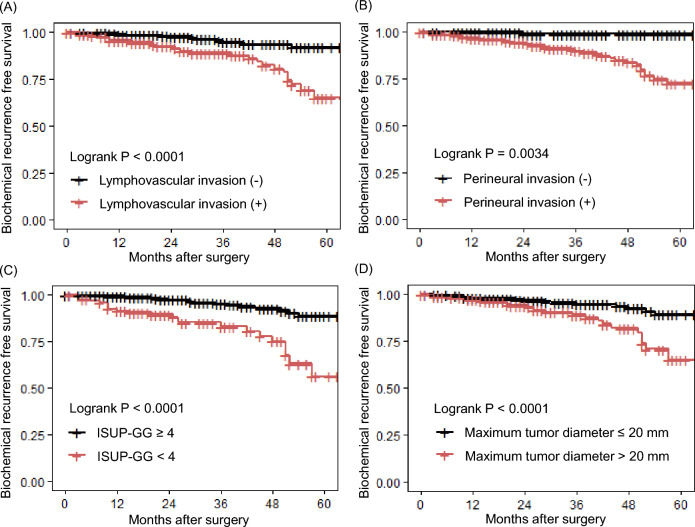
Kaplan–Meier survival curves in groups stratified by each factor of biochemical recurrence in patients with negative surgical margin after robot-assisted radical prostatectomy. *Lyv* lymphocascular invasion, *Pn* perineural invasion, *ISUP-GG* International Society of Urological Pathology grade group, *MTD* maximum tumor diameter, *BCRFS* biochemical recurrence free rate, *RARP* robot-assisted radical prostatectomy.

Univariate and multivariate analyses of factors predicting BCR in the experimental cohort are shown in Table 2. We identified ‘ISUP-GG ≥ 4’ (HR 3.20, 95% CI 1.70–6.03, P < 0.001), ‘perineural invasion’ (HR 10.7, 95% CI 1.45–79.9, P = 0.020), ‘presence of lymphovascular invasion’ (HR 2.03, 95% CI 1.00–4.12, P = 0.049), and ‘MTD > 20 mm’ (HR 1.9, 95% CI 1.01–3.70, P = 0.047) as independent predictors of BCR in the multivariate analysis (Table 2). Kaplan–Meier curves were drawn regarding these parameters and log-rank tests were performed for statistical analysis (Fig. 2A–D).

**Table 2:** Risk analysis of biochemical recurrence incorporating variables from final pathology in RARP patients with negative surgical margin (N = 537).

Variable	Univariate			Multivariate		
	Hazard ratio	95% CI	P value	Hazard ratio	95% CI	P value
PSA (≥ 20 vs. < 20 ng/mL)	1.01	0.97–1.05	0.611			
Prostate weight (< 40 vs. ≥ 40 g)	0.99	0.97–1.01	0.300			
ISUP-GG (≥ 4 vs. < 4)	4.79	2.58–8.89	< 0.001	3.20	1.70–6.03	< 0.001
Lymphovascular invasion (positive vs. negative)	3.98	2.03–7.80	< 0.001	2.03	1.00–4.12	0.049
Perineural invasion (positive vs. negative)	19.52	2.68–142.1	0.003	10.7	1.45–79.9	0.020
Extra prostatic extension (positive vs. negative)	2.10	1.11–3.97	0.020	1.0	0.51–1.95	0.995
Seminal vesicle invasion (positive vs. negative)	1.69	0.41–7.02	0.500			
MTD (> 20 vs. ≤ 20 mm)	2.89	1.54–5.43	0.001	1.9	1.01–3.70	0.047
MTD-mGS (> 20 vs. ≤ 20 mm)	2.85	1.53–5.33	0.001			

*RARP* robot-assisted radical prostatectomy, *PSA* prostate specific antigen, *CI* confidence interval, *ISUP-GG* International Society of Urological Pathology grade group, *max.* maximum, *MTD-mGS* maximum diameter of tumor with maximum Gleason score, *MTD* maximum diameter of tumor with any Gleason score.

### Risk model predicting biochemical recurrence in patients with negative surgical margin

**Figure 3:**
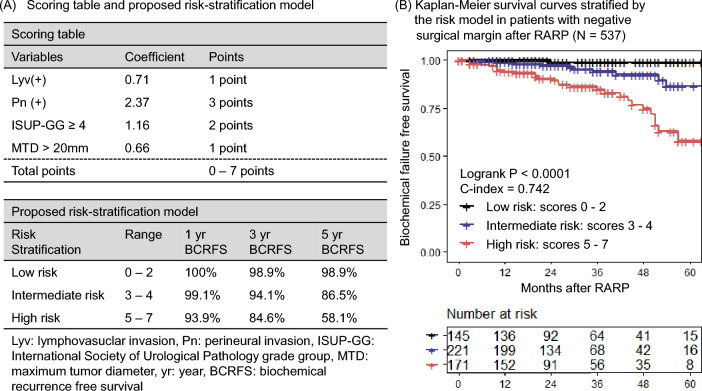
(**A**) Scoring table and the proposed risk-stratification model. Weighted scores were given according to the coefficient value in the multivariate analysis; 1 point is given if the value of the coefficient is 0–1, 2 points if the coefficient value is 1–2 and 3 points when it is over 2. Total scores were calculated and classified into three distinctive risk groups according to the total scores (0–2: Low risk, 3–4: Intermediate risk, 5–7: High risk). (**B**) Kaplan–Meier survival curves in each risk groups stratified by the proposed risk-stratification model in patients with negative surgical margins after robot-assisted radical prostatectomy.

**Figure 4:**
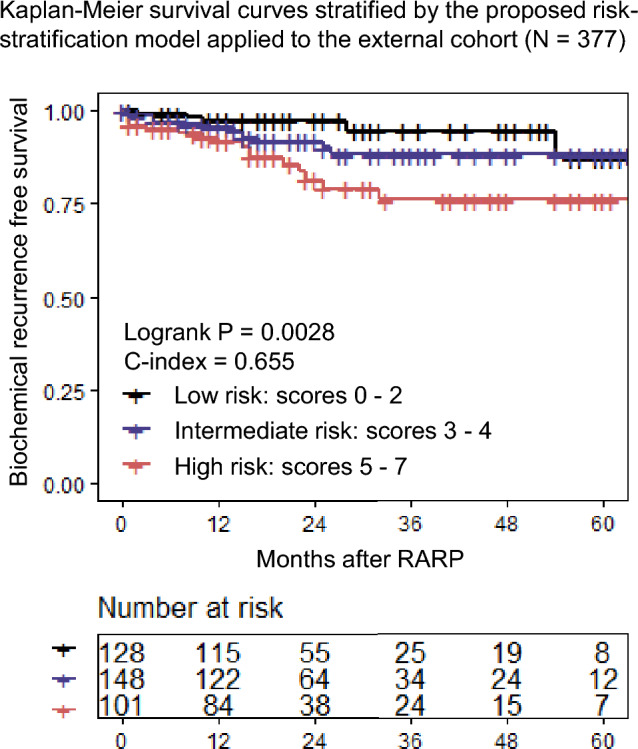
Kaplan–Meier survival curves in each risk group stratified by the risk-stratification model applied to the external cohort. *RARP* robot-assisted radical prostatectomy.

A risk stratification model for patients with negative surgical margin after RARP for nonmetastatic PCa was developed using 4 robust risk factors (‘ISUP-GG ≥ 4’, ‘presence of lymphovascular invasion’, ‘presence of perineural invasion’, and ‘maximum diameter of the tumor > 20 mm’) associated with BCR according to the multivariate analysis. Points were given according to the value of coefficients in the multivariate analysis. As a result, 3 points were given if ‘perineural invasion’ was present, 2 points were given if ‘ISUP-GG ≥ 4’ was present, and 1 point was given when ‘lymphovascular invasion’ or ‘MTD > 20 mm’ was present (Fig. 3A). The risk model exhibited statistical significance and good discrimination with a concordance of 0.742 (apparent c-index, Fig. 3B). We further performed an internal validation using the bootstrap method and the optimism-corrected c-index was 0.731. Based on this risk model, 1-year, 3-year, and 5-year BCR free survival (BCRFS) were 100%, 98.9%, 98.9% in the low-risk group; 99.1%, 94.1%, 86.5% in the intermediate-risk group; 93.9%, 84.6%, 58.1% in the high-risk group (Fig. 3A,B). The external validation was also performed and the c-index of this model was 0.655 (Fig. 4).

We also created a nomogram predicting BCRFS by using the same predictors (Supplementary Fig. 1A). This nomogram showed reasonable calibration results in 1, 3, and 5 years after RARP (Supplementary Fig. 1B–D).

## Discussion

In the present study, we investigated the clinical significance and risk factors of BCR in patients undergoing RARP with negative SM and established a novel risk model to predict BCR. We identified ‘ISUP-GG ≥ 4’, ‘perineural invasion’, ‘presence of lymphovascular invasion’, and ‘MTD > 20 mm’ as independent predictors of BCR in patients with negative SM. By using these parameters, we developed a novel risk model with good discrimination.

It is well known that positive SM is one of the strongest risk factors of BCF^4,5^. In theory, a patient with a large amount of remnant tumor may have early BCR whereas another patient with a small amount or no existence of remnant tumor may develop delayed recurrence or may not even develop recurrence at all. The status of positive SM depends on intraoperative surgical conditions that are different among studies^10–13^. This results in a wide range of BCR rates from 10 to 40%^10–13^. Reflecting this background, statistical analysis should be carried out separately in patients with negative and positive SM when analyzing factors associated with BCR to maintain universal validity in statistical analysis and to mitigate the impact of SM status. Indeed, many risk models and nomograms are predicting BCR in RARP patients^2,14^. Nevertheless, studies that analyze predictors of BCR using stratification of patients by SM status and developing a risk stratification model in such patients are rare. Moreover, due to the nature of heterogeneous characteristics of studies associated with surgery when compared with the rather homogeneous nature of studies on pharmaceuticals, this study is notable since it showed acceptable discrimination even in other independent external cohorts.

One theory of the biology of BCR in patients with negative surgical margin status is the possibility of micro-metastasis. Indeed, micro-lymphatic invasion and perineural invasion were significant predictors of BCR in the present study and these factors may theoretically contribute to micro-metastasis. Specifically, perineural invasion had the most significant impact in the present study. Several studies suggest its clinical significance in PCa^15,16^. From a biological perspective, current studies postulate that certain types of adhesion molecules, growth factors, and chemokines are involved in perineural-invasion associated tumor progression^17,18^. Tumor cells with perineural-invasion features can also up-regulate nerve growth, thereby increasing neurite quantity, axon lengthening, and density of nerve tissues^19^. As a result, new nerves in the tumor are developed that are capable of inducing immune escape of the tumor cells^19^.

PCa has a distinct characteristic regarding the TNM staging system since it does not have any description regarding the actual size of the tumor in the T stage, while the size of a tumor is an index parameter for staging in many other malignancies^20^. Hansen et al. reported that the percent tumor volume did not improve the prediction of early BCR after RP^21^. Another paper reported that maximum tumor diameter was not an independent prognostic factor in high-risk localized PCa^22^. However, recent studies have indicated the underestimated importance of tumor size in terms of prognosis and its clinical significance is gradually gaining acceptance as a significant predictor of BCF in PCa^23–25^. For instance, high percent tumor volume, which was defined as the calculation of the summed regions of interest (ROI) of all tumor lesions by the estimated volume of the resected prostate, was a predicting factor of BCR in RP patients^25^. After it became apparent that MTD increases in proportion to the actual tumor volume in patients with PCa^26^, even a simple measured diameter of the maximum-sized tumor was shown to be a significant factor of BCR. We previously reported MTD as a promising predictor of BCR in patients undergoing open radical prostatectomy^27^. In that study, BCR rates were 11% for tumors with maximum tumor diameter (MTD) of 0.9–10 mm but were three times as much for tumors with MTD of 21–30 mm. Another study showed that longer MTD was a significant risk factor for PSA failure in 354 patients who underwent RP for cT1c or cT2 PCa^28^. In the present study, MTD was a significant factor of BCF in patients with negative SM.

There is only one study evaluating risk factors predicting BCF in patients having negative SM after RARP^29^. This study by Hashimoto et al. showed that pathologic Gleason score ≥ 4 + 3 and micro-lymphatic invasion were significant factors of BCF in organ-confined PCa patients with negative SM after RARP^29^. Unfortunately, their study was limited by the lack of tumor volume status of the surgical specimen, which may have been imprecise to create a risk model. Additionally, the proposed risk stratification model was not validated internally or externally.

This study has limitation since it was conducted retrospectively. However, the strength of this study is that the risk model we suggested was evaluated by an external validation model using an integrated database of 3 individual institutions. Another limitation included the diagnosis of the pathological parameters used in this study since it was diagnosed by multiple pathologists in multiple institutions. Additionally, there is missing consensus of defining perineural invasion^30^. Given this, the prevalence of perineural invasion may be influenced to a certain degree.

In conclusion, we identified predictors of BCR and developed a risk model in patients with negative SM. The present study may support surgeons regarding decision-making on surgical indication and determination of follow-up-period.

### Supplementary Information

Supplementary Legends.

Supplementary Figure S1.

## Data Availability

The dataset used in the present study is not publicly available due to the ongoing clinical studies based on the same dataset. However, it can be used by a reasonable request to the corresponding author.
