# Supplementary material for: Development of risk-score model in patients with negative surgical margin after robot-assisted radical prostatectomy
Source: Sci Rep. 2024 Mar 31;14:7607. doi: 10.1038/s41598-024-58279-1 (PMC10982299; doi:10.1038/s41598-024-58279-1)
Supplement: Supplementary file 1 — Supplementary Legends. [file 41598_2024_58279_MOESM1_ESM.docx]

**Supplementary figure 1**

1. A nomogram predicting BCR after RARP based on the experimental cohort.

(B)-(D) Calibration plot validating the nomogram at 1, 3, and 5 years.
