# Supplementary material for: Development of risk-score model in patients with negative surgical margin after robot-assisted radical prostatectomy
Source: Sci Rep. 2024 Mar 31;14:7607. doi: 10.1038/s41598-024-58279-1 (PMC10982299; doi:10.1038/s41598-024-58279-1)

Fig.S1

(A) Nomogram predicting BCR after RARP

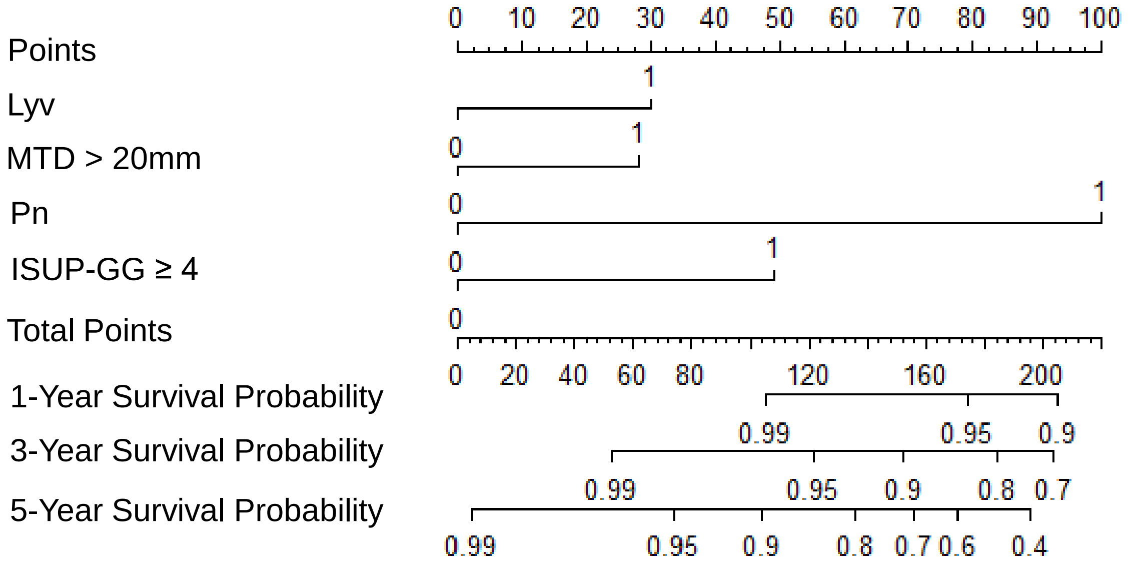

Abbreviations
BCR: biochemical recurrence
RARP: robot-assisted radical prostatectomy
Lyv: lymphovascular invasion
Pn: perineural invasion
ISUP-GG : International Society of Urological Pathology grade group

(B) Calibration plot at 1 year after RARP

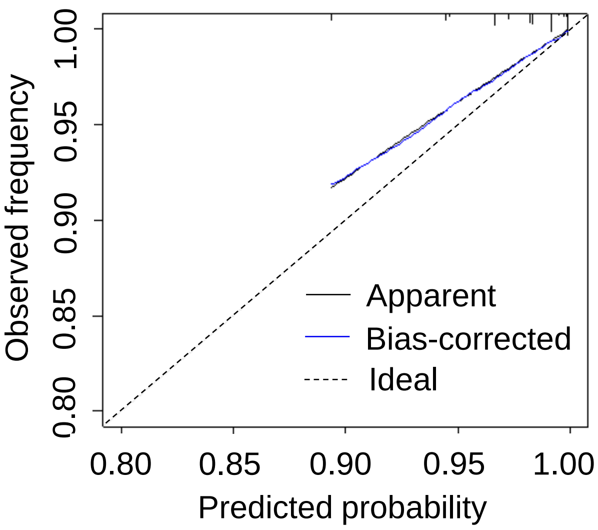

(C) Calibration plot at 3 years after RARP

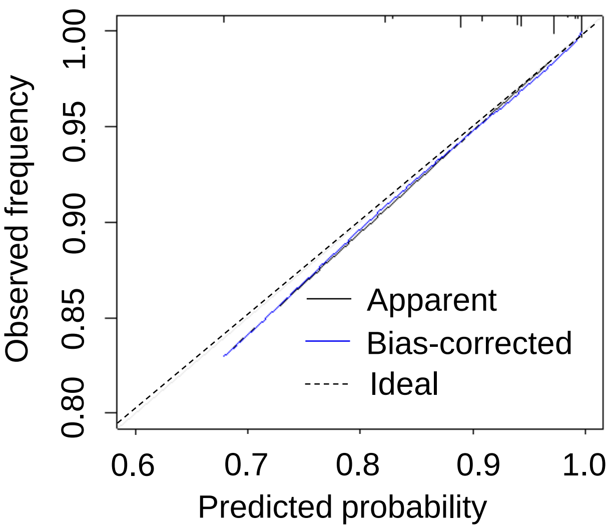

(D) Calibration plot at 5 years after RARP

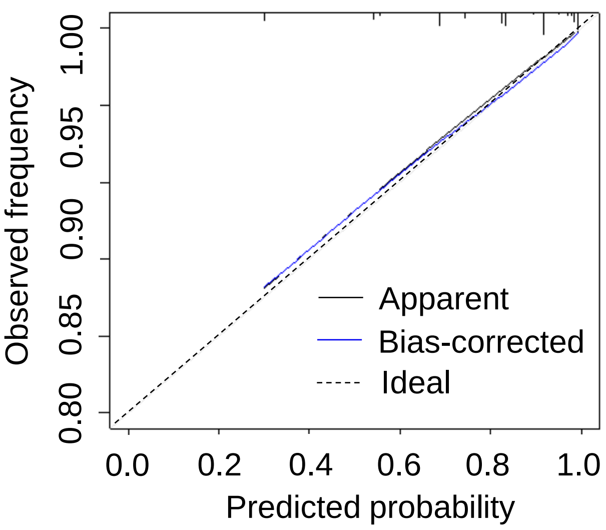

Supplement: Supplementary file 2 — Supplementary Figure S1. [file 41598_2024_58279_MOESM2_ESM.pdf]
